# Invasive Aspergillosis and the Impact of Azole-resistance

**DOI:** 10.1007/s12281-023-00459-z

**Published:** 2023-03-18

**Authors:** Davide Bosetti, Dionysios Neofytos

**Affiliations:** Division of Infectious Diseases, Geneva University Hospitals, Rue Gabrielle-Perret-Gentil 4, Geneva, Switzerland

**Keywords:** Invasive aspergillosis, Azole-resistance, Cryptic Aspergillus species

## Abstract

**Purpose of Review:**

IA (invasive aspergillosis) caused by azole-resistant strains has been associated with higher clinical burden and mortality rates. We review the current epidemiology, diagnostic, and therapeutic strategies of this clinical entity, with a special focus on patients with hematologic malignancies.

**Recent Findings:**

There is an increase of azole resistance in *Aspergillus* spp. worldwide, probably due to environmental pressure and the increase of long-term azole prophylaxis and treatment in immunocompromised patients (e.g., in hematopoietic stem cell transplant recipients). The therapeutic approaches are challenging, due to multidrug-resistant strains, drug interactions, side effects, and patient-related conditions.

**Summary:**

Rapid recognition of resistant *Aspergillus* spp. strains is fundamental to initiate an appropriate antifungal regimen, above all for allogeneic hematopoietic cell transplantation recipients. Clearly, more studies are needed in order to better understand the resistance mechanisms and optimize the diagnostic methods to identify *Aspergillus* spp. resistance to the existing antifungal agents/classes. More data on the susceptibility profile of *Aspergillus* spp. against the new classes of antifungal agents may allow for better treatment options and improved clinical outcomes in the coming years. In the meantime, continuous surveillance studies to monitor the prevalence of environmental and patient prevalence of azole resistance among *Aspergillus* spp. is absolutely crucial.

## Introduction 

The mold *Aspergillus* spp. was identified by an Italian priest Micheli in 1729, who named it after the shape of an Aspergillum (sprinkler of holy water) [1]. The classification of this mold is quite challenging. Since many informal classifications with no international consensus in the last years were proposed, such as “species complexes “ or “cryptic species,” the use of the classical taxonomy according to their morphology and phylogenetic relationships (6 subgenera, 27 sections, and 87 series) seems to be more reasonable [2, 3, 4]. Invasive aspergillosis (IA) is the most common invasive fungal disease (IFD) caused primarily by *A. fumigatus,* followed by *A. flavus*, *A. terreus,* and *A. niger*. [5] Prolonged neutropenia, hematopoietic cell transplantation (HCT), intensive chemotherapy, graft-versus-host disease (GVHD), and high-dose corticosteroids are classical risk factors for IA [6]. In the last decades, the development of new therapies, such as inhibitors of tyrosine kinase (e.g. ibrutinib) or Janus-kinase, checkpoint-inhibitors, and CAR-modified T-cells (due to the use of steroids and tocilizumab for the prevention of cytokine release syndrome), has also been associated with higher risk for IA [7, 8, 9]. Among solid organ transplant (SOT), lung transplant recipients have the highest risk of IA. [10, 11]

Furthermore, patients in the intensive care unit patients or infected with SARS-CoV-2 and patients with advanced liver cirrhosis or under long systemic corticosteroid therapy are also at risk for IA. [12, 13, 14]

Considering the increasing number of patient populations at risk for IA, higher numbers of patients are exposed to broad-spectrum azoles, such as voriconazole, posaconazole, or isavuconazole, either as prophylaxis or treatment [15, 16, 17, 18, 19]. However, prolonged and expanded use of broad-spectrum azoles in clinical practice, as well as in the environment, might have contributed to an important increase in azole resistance worldwide [20, 21]. Azole-resistant *Aspergillus* strains have been associated with therapeutic failure and mortality rates as high as 90% [22]. In this article, we review the current trends in the epidemiology and clinical impact of antifungal-resistant *Aspergillus* spp.

## Epidemiology of Azole-Resistant *A. fumigatus*

**Fig. 1:**
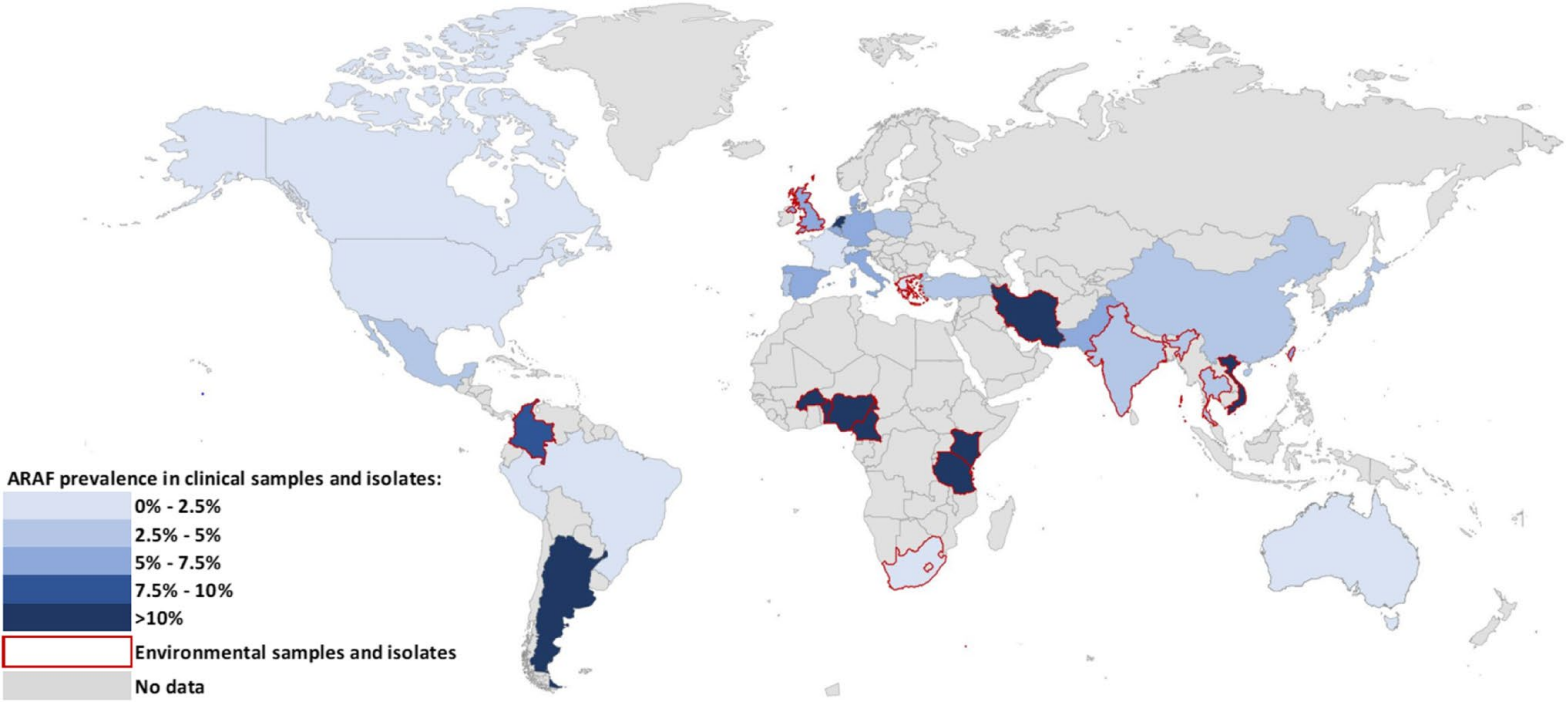
Worldwide prevalence of azole resistant *Aspergillus fumigatus* (ARAF) clinical and environmental samples and isolates

Azole-resistant *A. fumigatus* (ARAF) strains caused by the mutations TR34/L98H or TR46/Y121F/T289A have been reported worldwide [23]. The first case of ARAF was reported in the late 1980s in the Netherlands, and since then, increased rates of azole resistance have been reported in all continents, except for Antarctica (Fig. 1) [24]. Due to variability in agricultural use of azole-containing pesticides, scarce routine surveillance programs, and limited availability of routine susceptibility testing, there is a lack of relevant, detailed epidemiological data [25]. This could explain, in part, the enormous variability of ARAF prevalence in different countries [26]. The SCARE-Network, a multicenter study from 22 centers (19 European and 4 non-European sites), showed an overall ARAF-prevalence of 3.2% (ranging from 0 to 26%), with the predominance of the mutation TR34/L98H between 2009 and 2011 [26]. The Netherlands have reported one of the highest ARAF-prevalence in Europe, with a significant increase from 1.7–6% in the period 1997–2007 to 8–15% between 2013 and 2018 (TR34/L98H and TR46/Y121F/T289A mutations accounting for most cases) [27, 28]. In Denmark, ARAF prevalence was reported to be 6.1%, based on a national surveillance program between 2018 and 2020 [29]. A Belgian one-year retrospective multicenter study showed an ARAF prevalence of 5.5% [30]. In Spain, the estimated prevalence is between 1.2 and 6.6% [31, 32]. Study groups from Germany and Italy reported similar rates of azole-resistant *A. fumigatus* in cystic fibrosis patients, as well as in patients with hematological malignancies, 5.3–9 and 1.1–1.3%, respectively. [33, 34, 35, 36] Lazzarini et al. showed an azole resistance prevalence of 6.25% in Italian clinical samples [37]. Similarly, in France ARAF has been reported in 0.85% of hematological malignancy patients and 1.8% in the general population [38, 39]. In Portugal, Poland, and Turkey, the resistance rates in clinical isolates and samples were 2.6, 4.1, and 3.3% respectively. [40, 41, 42] Environmental isolates were reported to be azole resistant in 6% of cases in the UK and 1% of cases in Greece [43, 44]. In Switzerland, the TR34/L98H mutation was first reported in 2018, in environmental *A. fumigatus* strains initially, and later in two patients with cystic fibrosis [45]. Based on a recently published surveillance study on ARAF in clinical samples in Switzerland, ARAF prevalence was found to be about 1.1%. [46]

In the USA, the first reported TR34/L98H mutation was reported in 2016 [47]. Between 2015 and 2017 a passive surveillance program showed an ARAF prevalence of 1.4% [48]. Canada seems to have for the moment low rates of azole resistance, with a prevalence in clinical samples of 0.2%. In contrast, in Latin America, data are scarce. A Peruvian prospective cohort study in patients with chronic pulmonary aspergillosis showed a resistance prevalence of 2%, and a 1% resistance prevalence was recently reported in Brazil [49, 50]. Environmental isolates from Colombia showed a resistance prevalence of 9.3% [51]. In Mexico and Argentina, recent studies reported a resistance prevalence of 4.7% and 14%, respectively. [52, 53] In the African continent, data are also limited. Some data are suggesting ARAF prevalence of 1.3% in clinical samples and up to 17.1% in the environmental setting [54]. Resistance prevalence in Vietnam (environmental samples), Iran (environmental samples), China, Taiwan, Pakistan, Thailand, Japan, and India were 65, 18, 4.4, 7.5, 6.6, 3.2, 2.9, and 4.3%, respectively. [22, 23, 55, 56, 57, 58, 59, 60, 61, 62] In Australia, only 2% of clinical isolates of *A. fumigatus* were reported to be azole resistant [63]. In South Korea, no resistance strains were found until 2015. [64]

## Resistance in *Aspergillus fumigatus* “Cryptic” Species

The section *A. fumigatus* includes “cryptic” species causing IA, albeit associated with higher mortality rates [65]. “Cryptic” species are probably underestimated, since the conventional diagnostic methods are not often able to recognize them, and only DNA sequencing permits accurate identification and classification. The resistance mechanisms and specific mutations are mostly still unknown for these species, but there are some homologies with *A. fumigatus*. [65] The most frequent cryptic *A. fumigatus* species is *A. lentulus,* first described in 2004 in allogeneic HCT recipients [66]. It is mostly found in immunocompromised patients with hematological disease, SOT recipients or patients treated with high-dose systemic corticosteroids [67]. Although *A. lentulus* represents < 3% of all *A. fumigatus* spp. in clinical specimens, it could account for 10–30% of misidentified/sub-categorized ARAF [65, 68]. Mutations in the Cyp51A gene (as for *A. fumigatus*) are probably responsible for the development of azole resistance in *A. lentulus*. [69] Since this species has a low susceptibility to multiple antifungals, including azoles and echinocandins, treatment options are often limited [68]. *A. (Neosartorya)udagawae*, isolated for the first time in 1995, accounts only for a small percentage of IA cases but, as *A. lentulus,* is often resistant to voriconazole and amphotericin B [65, 70]. Other rare “cryptic” species in the section *fumigatus* include *A. viridinutans/felis* and *A. fischeri/thermomutatus*. [65]

## Epidemiology of Azole-Resistant Non-*fumigatus* Aspergillus Species

A large Spanish study showed a high prevalence of non-*fumigatus* species in clinical samples (*A. fumigatus* accounted for less than 50%) [31]. Overall, *A. flavus, A. terreus, A. tubigensis, A. nig*er, and *A. nidulans* represented 8.4, 8.1, 6.8, 6.5, and 2.5%, respectively, of all clinical samples in this population-based survey [31]. On the contrary, an American study in transplant recipients reported a higher prevalence of *A. flavus* (13.3%), followed by *A. niger* (6.0%) and *A. terreus* (5.0%) [71]. The prevalence of “cryptic” species between all identified *Aspergillus* sections was of 10% and 12% in the two previously cited studies, respectively. [31, 71] *A. flavus* is worldwide the second cause of IA after *A. fumigatus*, with higher prevalence reported in sub- and tropical countries like India, Pakistan, and Sudan [72, 73, 74, 75]. For instance, in India, 47% of all IA in ICU patients with no classical risk factors were caused by *A. flavus* (39.4% *A. fumigatus*) [75]. Azole resistance has been rarely reported in *A. flavus* strains [72]. *A. terreus* accounts for a minority of IA infections, but with a prevalence as high as 30% among IA cases in specific geographical regions such as Houston, Texas (USA) and Innsbruck, Tyrol (Austria) [76, 77]. Recent epidemiological data from Italy suggest a prevalence of *A. terreus* in hematological patients of 4.8% [36]. Posaconazole resistance has been found in 13.7% of all *A. terreus* isolates in Germany, 12.5% in the UK, and 10% in Austria, worldwide. A total of 5.4% of all section Terrei isolates were posaconazole-resistant; voriconazole and itraconazole resistance was rare and mostly found in the “cryptic” species of this section such as *A. citrinoterreus* and *A. alabamensis*. [78] Since the MICs for voriconazole (2 microgramm/mL) are higher than posaconazole (0.25 microgramm/mL) for this section, resistance to voriconazole is rare. Azole-resistance is extremely important for this species, due to the limited alternative treatment options for *A. terreus,* considering the amphotericin-B resistance, which is likely related to drug uptake reduction and higher catalase activity [79]. *A. citrinoterreus* is reported to be the most common « cryptic» species in the section *Terreus* (8.4%), followed by *A. hortai* (2.6%) and *A. alabamensis* (1.6%) [78]. Azole resistance remains rare in the cryptic species of the Terrei Sect.  [78] *A. niger* is considered to be of lower virulence in comparison to other *Aspergillus* spp. with incidence in transplant recipients between 0.048 and 0.16%, as reported by the TRANSNET group [80, 81]. A Belgian single-center study has showed a small number (16 cases) of IA caused by *A. niger* complex over a 7-year period [82]. The “cryptic” species *A. tubingensis* was responsible for 5 of these cases [82]. *A. tubingensis* as *A. awamori*/welwitschiaceae is known to have higher triazole MICs limiting the available treatment options [83]. *A. ustus* with the “cryptic” species *A. calidoustus* and *A. pseudodeflectus* are rare causes of IA, with a recent literature review showing 67 reported cases of probable/proven IA worldwide due to *A. ustus* complex [84]. Half of these cases were breakthrough IA in patients receiving azole-based antifungal prophylaxis, consistent with other reports suggesting increasing detection of this pathogen in the setting of long-term azole prophylaxis [84, 85]. Since resistance to azole of these species is variable, rates of therapeutic failure and mortality are high [84]. Also, *A. nidulans* with the “cryptic”species *A. sublatus*, *A. quadrilineatus* is a very rare cause of IA, described mostly in patients with the chronic granulomatous disease (estimated to cause 35% of all IA in this patients subgroup), observed in osteomyelitis and in patients receiving itraconazole prophylaxis [86, 87, 88]. Azole resistance is not common in the section *A. nidulans*. [87]

## Mechanisms of Azole and Other Antifungal Resistance

The pathogenesis of azole resistance is complex and multi-factorial. Moreover, most of the studies have been conducted in *A. fumigatus*, and data are missing for other species. More studies are needed, considering the important clinical burden of these infections [89]. First, single point mutations in the cyp51 gene (mostly amino-acid substitutions in CYP51A, such as G54, G138, M220, G448, L98H, Y121F, and T289A) can reduce the affinity between azoles and their targets [90, 91, 92]. These mutations are often associated with long-term azole administration [22]. For instance, mutations in the cyp51C gene are associated with high voriconazole minimal inhibitory concentrations (MIC) in *A. flavus*. [93, 94] Furthermore, cyp51A gene mutations may probably be the reason for *A. lentulus* azole-resistance [65]. Second, tandem repeat mutations of 34 bp (TR34), 46 bp (TR46), or 53 bp (TR53) in the promoter region of *A. fumigatus* cyp51A or other type of mutations in different genes could possibly induce an overexpression of cyp51, which increases the azole concentrations needed to inhibit fungal growth (since azoles are binding to cyp51 protein in order to block the formation of ergosterol) [95, 96, 97]. This type of mutation is thought to be associated with environmental azole resistance, since the massive use of azole-based fungicides in agriculture could provoke cross-resistance with triazoles [24, 98]. Such mutations have been described in azole-naive, but also in patients on long-term azole treatment, highlighting the complexity of the different types of resistance mechanisms [99]. Finally, multidrug efflux pumps could lead to lower intracellular drug concentrations and azole resistance, although more data are required to better describe this resistance mechanism [97, 100, 101]. Other possible mechanisms include cellular stress response, drug enzymatic degradation, and bio-film formation. [92, 102]

## Diagnosis of Azole-Resistance

The diagnosis of azole resistance is based on the phenotypic measurement of MIC, which is the threshold to differentiate a resistant from a susceptible strain. MICs are used together with pharmacokinetic/pharmacodynamic (PK/PD) data to determine the clinical breakpoints (CBPs), which are susceptibility predictors [103, 104, 105]. The gold standard to assess the MIC is the broth microdilution assays standardized by the Clinical and Laboratory Standards Institute (CLSI) or the European Committee on Antimicrobial Susceptibility Testing (EUCAST), which are the two major classification systems used for antimicrobial susceptibility [103, 104, 105]. Of note, EUCAST has established CBPs for different drugs for *A. fumigatus, A. flavus, A. nidulans, A. niger*, and *A. terreus*, while CLSI has proposed only voriconazole CBPs for *A. fumigatus*. [105] Otherwise, CLSI uses epidemiological cut-off values (ECVs) to differentiate among wild-type susceptible and non-wild-type strains, which are not predictors of clinical outcomes [106]. Overall, CLSI and EUCAST recommendations are similar, despite methodological differences [107]. Other complementary phenotypic diagnostic tests include calorimetric endpoint or agar-based methods that use MIC strips. These tests are easier to perform and comparable to the EUCAST/CLSI microdilution standard, but they also need a positive culture. [1, 26]

Genotypic testing for azole resistance is based mostly on PCR methods, targeting directly the most frequent point mutations in the cyp51A gene and its promoter and the tandem repeat insertions [1, 25, 108]. Currently, there are three different commercial kits used in Europe for the genotypic detection of azole resistance [109]. However, not all mutations are reported; hence sensitivity remains low and those PCR assays target only *A. fumigatus*-related mutations [1]. Pyrosequencing can also be used for detecting resistance, using the detection of light that is released during nucleotide incorporation into the amplifying DNA, but it is not often available [110]. MALDI-TOF could be also used for detecting azole resistance, but data on molds are largely missing [111]. Eventually, whole genome sequencing (WGS) has the highest resolution for detecting mutations in *A. fumigatus* and could be used for different other species. Due to high costs, long turn-around time, and high-level expertise needed, its utility remains very limited. [1, 112]

## Special Clinical Considerations for Hematopoietic Stem Cell Transplant Recipients and Patients with Hematologic Malignancies

The incidence of IA in patients with acute myeloid leukemia or allogeneic HCT recipients has ranged between 5 and 15%. [6, 113, 114]

Allogeneic HCT recipients with IA due to azole-resistant *Aspergillus* spp. present clinically similar to patients infected with non-resistant strains, usually with a pulmonary infection, followed by sinusonasal and cerebral IA [115]. Rapid detection of azole resistance is fundamental, since infections caused by these strains are associated with higher mortality, between 88 and 100% [116, 117, 118]. Comparative studies between azole resistant and susceptible strains of *Aspergillus* spp. in IA showed a 21–31% higher mortality in the azole resistant group [116, 119]. As already mentioned, phenotypic diagnostic tests that require culture growth often miss “cryptic” species and the rate of culture-positivity varies between different patient groups [120, 121]. Clinical suspicion for resistant *Aspergillus* spp. should be rapidly raised in the context of a lack of clinical and biological (e.g., persistence of high galactomannan plasmatic levels) response to the administered therapy.

## Antifungal Treatment Considerations

The cornerstone of IA treatment is broad-spectrum triazoles, including voriconazole, isavuconazole, and posaconazole [15, 16, 122, 123, 124, 125]. Different factors, such as prior use of azole prophylaxis, local epidemiology of azole-resistance, co-morbidities, site of infection, the severity of clinical presentation, and co-infections with other fungi, may impact the choice of antifungal agent used, particularly before microbiological results become available [6, 126]. For azole-resistant *Aspergillus* strains, there are no controlled clinical trial data; hence treatment recommendations are based mostly on expert opinion [22]. Latest treatment recommendations suggest avoiding an azole as empirical treatment if the local azole-resistance prevalence is > 10% and consider using either amphotericin-B lipid formulations or a combination of voriconazole with an echinocandin [16, 127, 128, 129]. However, there is a lack of clinical evidence to support this approach, particularly considering that the cut-off of 10% of azole resistance is arbitrary. However, this approach may be justified, considering the lack of availability of rapid molecular diagnostic tools in most centers, which could lead to significant delays in appropriate treatment initiation. If resistance rates are less than 5%, resistance investigation may be warranted only in case of treatment failure; however, with a prevalence between 5 and 10%, routine resistance testing is recommended [13]. The right timing for transition to oral antifungal treatment, when susceptibility testing is not available, is unclear. The European Society of Clinical Microbiology and Infectious Diseases (ESCMID) guidelines strongly recommend for the treatment of voriconazole-resistant (*MIC* > 2 mg/ml) IA amphotericin-B lipid formulation monotherapy or a combination of voriconazole or isavuconazole with an echinocandin [14]. Even if the MIC of posaconazole is 0.5 mg/l, a possible step-down therapy with oral posaconazole with a targeted plasmatic level > 3.3 mg/l has been suggested as a possible treatment option. [130, 131, 132]

As mentioned above, resistance testing may not always be feasible, because of the lack of culture growth and isolation difficulties for cryptic species in specific patient subgroups. In these cases, diagnosis of possible resistance is predominately based on clinical suspicion and treatment should always be discussed with the local infectious disease team. The “cryptic” species for the section *A. fumigatus*, such as *A. udagawae*, *A. thermomutatus*, and *A. lentulus*, show high MICs for all azole drugs, and *A. lentulus* and *A. udagawae* have higher MICs also for amphotericine-B products [65, 133, 134]. Isavuconazole seems to be active against *A. lentulus* and *A. udagawae*, although data are very limited. [63]

Concerning the therapy for non-*fumigatus Aspergillus* spp., voriconazole is the first-line therapy for *A. flavus*. [70] Echinocandins could be used in combination with voriconazole or alone when no other treatment options are available in the rare cases of azole resistance [70]. Since posaconazole resistance is common for *A. terreus* and his “cryptic” species, voriconazole and itraconazole are the preferred antifungal molecules [16, 135]. Liposomal amphotericin-B should be avoided [135]. “Cryptic” species of the *A. niger* section (example given *A. tubigensis*) are often resistant to the triazoles; for this reason, liposomal amphotericine B is preferred [136]. The optimal antifungal therapy for *A. ustus* is still not elucidated due to the absence of clinical trials. ESCMID recommend the use of amphotericin-B products, since often these infections are breakthrough IA under posaconazole or voriconazole prophylaxis [14]. Isavuconazole, which shows lower MICs, could have a potential role in the therapy of this Sect.  [137]

New antifungal molecules could play a role in the treatment of azole-resistant IA in the future. For example, ibrexagungerp shows an activity against azole-resistant isolates of *A. calidoustus* and *A. terreus,* if used in combination with liposomal amphotericin-B or azole [138]. Rezafungin has displayed some activity against azole-resistant *Aspergillus* spp. in animal models [139]. Fosmanogepix, VL-2397, and olorofim have also demonstrated a potent activity against azole-resistant strains. 140, 141 While more clinical data are necessary to better define the role of those new molecules in the management of IA due to “cryptic” and azole-resistant *Aspergillus* spp., the thus far reported MIC data of some of them suggest that they may represent an important tool in the management of those infections.

## Conclusions

There is an increase in azole resistance worldwide probably due to environmental phenomena (massive use of azole-containing fungicide in agriculture) and due to the increase of long-term azole prophylaxis and treatment in immunocompromised patients. IA caused by those strains has been associated with higher clinical burden and mortality rates. Rapid recognition of resistant *Aspergillus* spp. strains is fundamental to initiate an appropriate antifungal regimen in allogeneic HCT recipients and patients with hematologic malignancies. Clearly, more studies are needed in order to better understand the resistance mechanisms and to optimize the diagnostic methods to identify *Aspergillus* spp. resistance to the existing antifungal agents/classes. Currently, therapeutic approaches are challenging, due to multidrug-resistance strains, drug interactions, side effects, and patient-related conditions. More data on the susceptibility profile of *Aspergillus* spp. against the new classes of antifungal agents, which are in phase I–III clinical trials, may allow for better treatment options and improved clinical outcomes in the coming years. In the meantime, continuous surveillance studies to monitor the prevalence of environmental and patient prevalence of azole resistance among *Aspergillus* spp. is absolutely crucial.
